# Importance of orthodontic intervention of the Class III malocclusion in mixed dentition

**DOI:** 10.1590/2177-6709.25.5.057-065.bbo

**Published:** 2020

**Authors:** Dennyson Brito Holder da Silva, Ariane Salgado Gonzaga

**Affiliations:** 1 Academia Norte-Rio-Grandense de Odontologia (Natal/RN, Brasil).; 2 Universidade Federal do Rio Grande do Norte, Departamento de Odontologia (Natal/RN, Brasil).

**Keywords:** Interceptive orthodontics, Corrective orthodontics, Malocclusion

## Abstract

**Introduction:**

Supervising the development of occlusion, managing problems during the transition from mixed to permanent dentition, as well as controlling environmental factors that contribute to establishing malocclusion, are important actions to achieve a Class I occlusion with facial balance. Among these problems, the malocclusions associated with dysfunctions such as mouth breathing or obstructive sleep apnea syndrome (OSAS), atypical swallowing and abnormal tongue position, open bites, crossbites and maxillomandibular discrepancies, and especially the Class III malocclusion can be listed.

**Objective:**

The purpose of this article is to present and discuss the main aspects relevant to the benefits of performing the treatment of Class III malocclusion in patients with growth.

## RESUMO

**Introdução:**

A supervisão do desenvolvimento da oclusão e o gerenciamento de problemas durante a transição da dentição mista para a permanente, bem como o controle de fatores ambientais que contribuem para estabelecer a má oclusão, são importantes ações para se obter uma oclusão de Classe I com equilíbrio facial. Entre esses problemas, pode-se considerar más oclusões associadas às disfunções como respiração bucal ou síndrome da apneia obstrutiva do sono (SAOS), deglutição atípica, posição anormal da língua, mordidas abertas e mordidas cruzadas e discrepâncias maxilomandibulares, especialmente, a má oclusão de Classe III.

**Objetivo:**

O objetivo do presente artigo é apresentar e discutir os principais aspectos pertinentes aos benefícios de se realizar o tratamento da má oclusão de Classe III em pacientes com crescimento.

## INTRODUCTION

Supervising the development of occlusion, managing problems during the transition from mixed to permanent dentition, as well as controlling environmental factors that contribute to establishing malocclusion, are important actions to achieve Class I occlusion with facial balance, which often does not occur naturally without interceptive orthodontic treatment. Orthodontic approaches may be related to different categories of problems, such as a malocclusion in development, in which it may be necessary to intervene to reduce or interrupt the unfavorable change;^1^ or a dentition whose normal development can be interrupted by some local etiological factor, which requires treatment to maintain or restore the appropriate development.^2^

Some of the most relevant objectives of supervising the development of occlusion are to properly manage the growth potential in order to intercept skeletal imbalances, eliminate functional deviations, improve self-esteem, minimize trauma and prevent periodontal problems.^3^ The possible advantages of the early intervention are the emotional satisfaction of the child, the growth potential available at this stage of development, greater collaboration with treatment, the possibility of a more simplified second phase and the possible reduction of extractions in the corrective phase of treatment. Disadvantages also exist, such as inefficiency, longer treatment time, immaturity of the patient, inefficient oral hygiene, inability to care for the devices and cost.

The ideal age to treat malocclusions in growing patients has been a widely discussed and controversial topic. One of the most important debates is to stop the development of problems with early treatment or to delay therapy. Among these problems, the malocclusions associated with disorders such as mouth breathing or obstructive sleep apnea syndrome (OSAS), atypical swallowing and abnormal tongue position, open bites and crossbites, and maxillomandibular discrepancies, and especially the Class III malocclusion can be listed.

Class III malocclusion is a condition that can be classified as dentoalveolar, skeletal or functional, and its etiology will determine the diagnosis and prognosis of treatment.^4^ This malocclusion must be intercepted early, preferably during the deciduous dentition phase, since Class III tends to exacerbate itself during growth, especially during adolescence.^4^ ^-^ ^6^ The sooner treatment is started, the greater the compensatory orthopedic effects of the inevitable orthodontic discrepancies, which can often prevent need for orthognathic surgery at the end of growth. In addition, the early treatment of Class III brings psychological benefits, due to the improvement of facial aesthetics that also implies in the improvement of self-esteem.^5^ ^,^ ^6^

Long-term studies of early treated Class III malocclusions reveal that the results of the treatment are stable, with visible improvement in facial profile, occlusion and masticatory functions.^4^ ^,^ ^6^ Maxillary protraction therapy with a facemask is the most common treatment for patients with skeletal Class III due to maxillary retrusion, as it stimulates maxillary advancement and assists in the control of mandibular development.^7^ As this type of treatment must be started early, the anchorage is performed on permanent and/or deciduous teeth, stimulating the movement of the maxilla forward, rotating the mandible down and back, and decreasing the rotation of the palatal plane. There is also the projection of the upper incisors, mesialization and extrusion of the upper molars and the retroinclination of the lower incisors.^4^ ^-^ ^7^

In addition to the anteroposterior skeletal discrepancy, it is common to find other malocclusions associated with Class III due to maxillary hypoplasia, such as posterior crossbite and anterior open bite. Once the muscular balance is compromised by the negative overjet, habits such as the anteriorization of the tongue on swallowing and phonation are perpetuated during the child's development, changing the muscle tone, the posture at rest, and consequently establishing the anterior open bite.^4^ ^,^ ^6^ ^,^ ^8^ ^,^ ^9^ It is for this reason that the interception of oral habits and multiprofessional treatment is essential for the stability of the results obtained with orthopedic and orthodontic therapy.^4^ ^,^ ^8^ ^,^ ^9^

Therefore, the objective of this article is to present and discuss the main relevant aspects of the benefits of carrying out the supervision of the development of the occlusion, in addition to describing the interceptor and corrective orthopedic and corrective treatment of a patient with growing Class III malocclusion (case report presented to the Brazilian Board of Orthodontics and Facial Orthopedics).

## CASE REPORT

Male patient, at the end of the first transitional period of mixed dentition, aged 8 years and 4 months, with good general health and without carious lesions or periodontal problems. During the initial consultation, the patient reported as the main complaint *“the lower part is crossed and developed”*, in addition to the practice of parafunctional habits.

**Figure 1:**
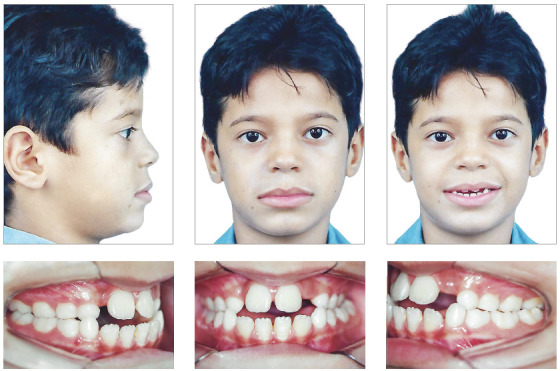
Facial and intraoral initial photographs.

Upon extraoral examination, the patient's face revealed typical characteristics of Class III malocclusion, with a deficiency of the middle third of the face, without zygomatic projection, showing the sclera in the lower part of the iris and active lip sealing. In frontal view, there was a slight facial asymmetry with mandibular deviation to the right, while in lateral view it showed a concave profile, with a chin-neck line apparently adequate to the face size (Fig 1). During the anamnesis and initial examination, the parafunctional habits of lingual interposition in phonation, adapted swallowing and tongue hypotonia were found.

**Figure 2:**
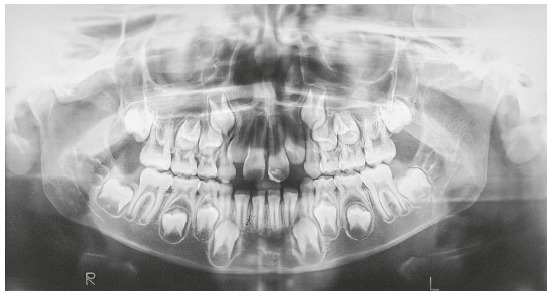
Initial panoramic radiograph.

The intraoral analysis showed an Angle Class I dental relationship, maxillary hypoplasia, bilateral posterior crossbite, anterior crossbite with a - 6 mm overjet, anterior open bite of 7 mm and inverted lower Spee curve. In addition, there was a severe lack of space of -8 mm in the upper arch to the lateral incisors irruption, biprotrusion and diastema between the upper central incisors. Despite the slight mandibular deviation to the right and the existence of diastemas, the upper and lower midlines were coincident (Fig 2).

**Figure 3:**
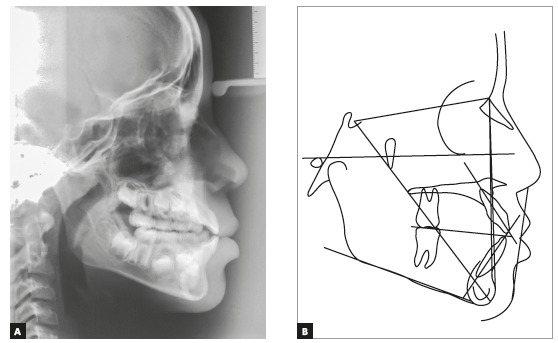
Initial cephalometric radiograph (A) and cephalometric tracing (B).

**Figure 4:**
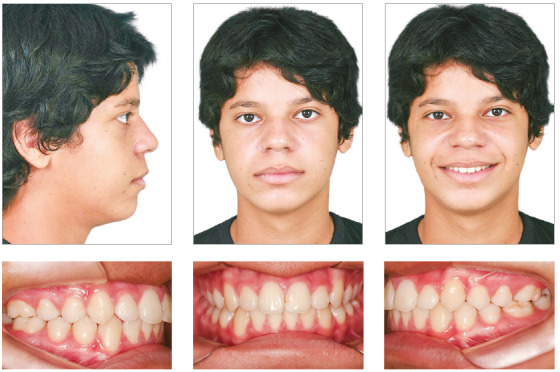
Facial and intraoral final photographs.

In the initial panoramic radiographic examination, it was observed the impaction of upper lateral incisors, with their roots in the developmental stage 8 of Nolla. The lack of space for the irruption of upper canines was also noticed, while the other permanent teeth had normal development and positioning (Fig 3). The lateral teleradiography of the face showed excessive vestibular inclination of the upper and lower incisors, maxillary hypoplasia, mandible with adequate size and position, and relatively short cranial base (Fig 4).

Steiner's cephalometric analysis revealed a growth tendency of Class III (SNA = 80°, SNB = 80° and ANB = 0°), while Wits^10^ analysis (- 4 mm) showed a real Class III. The patient had a horizontal growth pattern (Y axis = 54°, FMA = 22° and SN.GoGn = 31°) and dental biprotrusion confirmed by measurements 1.NA = 29°, 1-NA = 6 mm, 1.NB = 35°, 1-NB = 8mm and 1.1 = 114° (Table 1).

**Table 1:** Comparison of the initial and final cephalometric measurements of the patient.

	Measurement		Normal	A	B	Dif. A/B
Skeletal pattern	SNA	(Steiner)	82°	80°	80°	0
	SNB	(Steiner)	80°	82°	82°	0
	ANB	(Steiner)	2°	0°	2°	2
	Wits	(Jacobson)	♀ 0 ± 2mm	-4mm	+1mm	5
			♂ 1 ± 2mm			
	Angle of Convexity	(Downs)	0°	0°	2°	2
	Eixo Y	(Downs)	59°	54°	58°	4
	Facial Angle	(Downs)	87°	90°	88°	2
	SN.GoGn	(Steiner)	32°	31°	34°	3
	FMA	(Tweed)	25°	22°	25°	3
Dental pattern	IMPA	(Tweed)	90°	105°	102°	3
	1.NA (graus)	(Steiner)	22°	29°	27°	2
	1-NA (mm)	(Steiner)	4 mm	6mm	8mm	2
	1.NB (graus)	(Steiner)	25°	36°	35°	1
	1-NB (mm)	(Steiner)	4 mm	8mm	8mm	0
	- Interincisal Angle	(Downs)	130°	114°	114°	0
	1 - APg	(Ricketts)	1 mm	7mm	8mm	1
Profile	Upper lip-S line	(Steiner)	0	3mm	3mm	0
	Lower lip-S line	(Steiner)	0	5mm	4mm	1

## TREATMENT PLAN

A two-stage treatment was suggested due to the type of malocclusion. The first stage was the orthopedic treatment with palatal disjunction, maxillary protraction and interception of the parafunctional habit; and the second, the corrective orthodontic treatment with fixed appliances.

For the first phase of treatment, a modified Haas appliance was planned, with vestibular hooks, anchored on the deciduous second molars with a protocol of activation twice a day (morning and night), for 10 days or until overcorrection of the posterior crossbite. Petit's facemask was installed, with 500gF on each side and daily use of at least 16 hours. A lingual arch with spurs was also placed to intercept the tongue interposition habit. The maxillary protraction mechanics was actively conducted for approximately one year, a period necessary for the overcorrection of the anteroposterior discrepancy, and after this period, another six months of night use to preserve the results obtained. Spurs welded to the lingual arch were maintained during the second phase of treatment, until the correction of the anterior open bite, at which point the patient was referred for speech therapy.

The second phase of the treatment consisted of the corrective orthodontics, with the use of a Roth prescription (0.022 x 0.028-in) fixed metal appliance. A 4x2 mechanics was adopted in order to correct the Spee curve of the lower arch. After the initial alignment and leveling, Class III intermaxillary elastic mechanics (3/16-in, medium strength) and intercuspation mechanics with 1/8-in medium strength elastics in a 0.019 x 0.025-in braided stainless steel wire were applied. A removable wraparound retainer was planed for the upper arch, and a fixed 3x3 lingual bar, made with 0.018-in twisted flex wire, and maintained indefinitely for the lower arch.

## RESULTS

**Figure 5:**
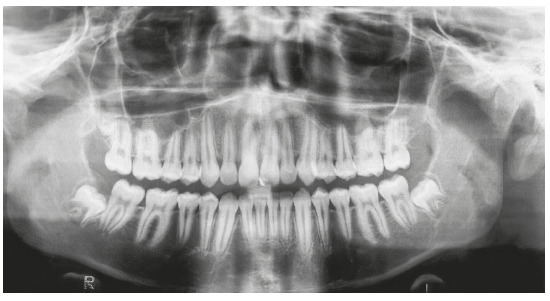
Final panoramic radiograph.

**Figure 6:**
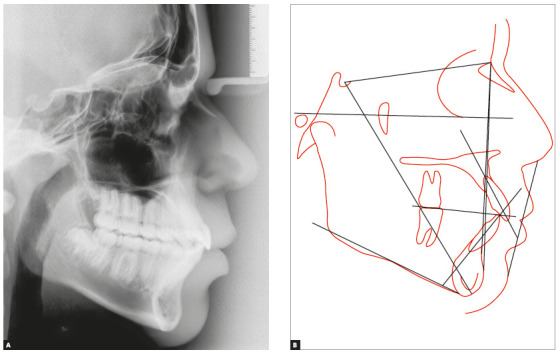
Final cephalometric radiograph (A) and final cephalometric tracing (B).

**Figure 7:**
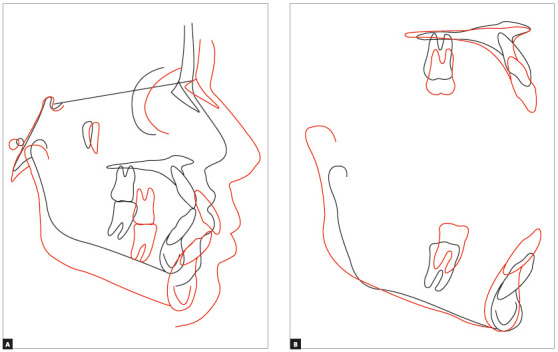
Total (A) and partial (B) overlays of the initial (black) and final (red) cephalometric tracings.

At the end of the treatment, the initial objectives were achieved, with a visible improvement in the facial profile and anteroposterior relationship of the face (Fig 5). A Class I of molars and canines was obtained, correction of the Spee curve, correct transversal relationship between the arches and adequate overbite and overjet (Fig 6). With the association of orthopedic and orthodontic mechanics it was possible to redirect the craniofacial growth, obtaining a Skeletal Class I relationship (ANB = + 2° and Wits = +1 mm) (Table 1, Figs 7, 8, 9 and 10). Thus, adequate functional guides and correct posture and tonus of the tongue were established.

## DISCUSSION

The assessment and treatment of occlusal and skeletal disharmonies can be initiated at various stages of development, depending on the severity, the pattern of skeletal growth, as well as the risks and benefits of treatment itself. Early treatment is definitely a viable possibility; however, it is not indicated for all patients. The objectives of the early orthodontic intervention include controlling unfavorable growth, preventing aggravation of dental and skeletal disharmony, improving occlusion and aesthetics of the smile. Therefore, it is recommended to supervise the development of the occlusion throughout the tooth eruption process in order to offer treatments with more predictable results. In this Class III clinical case, the interceptive approaches to deleterious oral habits together with the early orthopedic treatment of malocclusion, were determining factors for the treatment outcome.^11^ ^-^ ^21^

Some malocclusions, such as crossbites, do not correct themselves and tend to worsen during the child's growth and development. Therefore, they should be treated as soon as they are diagnosed. There are several reasons for starting treatment in the early stages of mixed dentition: taking advantage of bone bioelasticity; prevent joint disorders; redirect growth towards the normal development of facial and skeletal characteristics; prevent dental disharmonies from evolving to skeletal ones, and improve the breathing pattern in children with mouth breathing or OSAS. In this period, the correction of skeletal changes is simpler and with a lower biological cost for the patient, as in the case of correction of the posterior crossbite by means of disjunction of the median palatal suture. In children aged 8 to 10 years, this palatal suture is wide and with more regular edges, whereas in later periods of growth (10 to 13 years) this suture becomes more irregular and juxtaposed.^22^

The best moment to start treatment in patients with skeletal Class III associated with maxillary retrusion has been widely discussed by studies supported by clinical observations. The periods of primary dentition and the first transitional period of mixed dentition, around 6 years of age, are the most propitious to initiate maxillary protraction, since the orthopedic effects are more expressive, with significant advances in points A and ANS (anterior nasal spine)^5^ ^,^ ^23^. In these periods there is a greater predisposition to anterior displacement of the maxilla, increasing the growth in the maxillary and circummaxillary sutures, which are regular and wide before 8 years of age and become more strongly interdigited near puberty.^2^ In the initial stage of mixed dentition, the best orthopedic responses are observed in the correction of posterior skeletal crossbite,^24^ anterior open bite,^25^ and skeletal Class III.^1^

The therapeutic decisions made for the first phase guaranteed the results obtained at the end of the entire treatment. The indication of lingual spurs is presented in the literature as a valid therapeutic modality to eliminate the habit of interposing and reeducating the tongue posture, contributing to the correction of the anterior open bite.^11^ ^,^ ^15^ ^,^ ^26^ ^-^ ^30^ For this reason, immediately after the disjunction of the maxilla, the lingual arch with welded spurs was installed. So that, without the interference of the tongue, the correction of the anterior open bite occurred simultaneously with the effects of the treatment with the facemask that redirected the maxillary growth forward and down.

The decision for maxillary protraction was based on evidence proven by literature that the Class III treatment with the facemask is the most widely chosen for the correction of the retrognathic maxilla.^13^ ^,^ ^17^ Studies show significant favorable results in the correction of dental and skeletal variables, such as positive changes in the Wits analysis indexes and in the correction of the patient's overjet.^5^ ^,^ ^17^ ^,^ ^31^ These previously reported characteristics corroborate the results of this clinical case, which culminated in the improvement of Wits analysis values from -4 mm pre-treatment to + 1 mm after treatment, and adequate overjet and overbite.

The protocols adopted for maxillary protraction of this patient are also in accordance with those stated by the scientific literature, such as previous maxillary disjunction^5^ ^,^ ^17^ ^,^ ^31^ ^-^ ^33^ followed by protraction of the maxilla with an approximate direction of 30° downwards and forwards, and magnitude of force between 300gF and 600gF per activation side.^5^ ^,^ ^31^ ^-^ ^35^ This first phase of treatment promoted a more favorable environment for the expression of facial growth and development, correcting occlusal relationships, improving facial aesthetics and self-esteem, and minimizing permanent skeletal deformations in the adult phase.^19^

Therefore, the treatment of Class III performed during the growth period promoted positive results. However, the hereditary character of this malocclusion can compromise the results obtained with early treatment, making more invasive treatments such as orthognathic surgeries necessary, if the patient is not properly monitored orthodontically until the end of his growth. Thus, it is important that the treatment of Class III is carried out in two stages, the first stage of interception, with orthopedic and functional treatment, and the second stage, of orthodontic treatment with fixed appliances, for the refinement of occlusal relationships, with use of Class III intermaxillary elastics for the consolidation of Class I obtained after maxillary protraction therapy.^17^

## CONCLUSION

The intervention and supervision of skeletal Class III performed in patients before the growth spurt, associated with the interception of deleterious oral habits and effective and efficient orthodontic mechanics are decisive factors for the success of orthodontic treatment of this malocclusion.
